# Supplementary material for: Women Taking a Folic Acid Supplement in Countries with Mandatory Food Fortification Programs May Be Exceeding the Upper Tolerable Limit of Folic Acid: A Systematic Review
Source: Nutrients. 2022 Jun 29;14(13):2715. doi: 10.3390/nu14132715 (PMC9268323; doi:10.3390/nu14132715)
Supplement: Supplementary file 1 [file nutrients-14-02715-s001.zip › File S1 - detailed search strategy v2.pdf]

Permanent Searches 6

PERMANENT SEARCHES 6

Favorites Only

Run Copy Delete

Folate intake - intake with supps Permanent
Or

Medline OVID final_ 71021 Permanent
Countries, population (not humans, not adults, not female)folate, intake (food fortification etc)

1. Andorra/
2. "Antigua and Barbuda"/
3. Aruba/
4. Australia/
5. Austria/
6. Bahamas/
7. Bahrain/
8. Barbados/
9. Belgium/
10. Bermuda/
11. British Virgin Islands/
12. Brunei/
13. Canada/
14. West Indies/
15. Channel Islands/
16. Chile/
17. Croatia/
18. Curacao/
19. Cyprus/
20. Czech Republic/
21. Denmark/
22. Estonia/
23. Faroe Islands.mp.
24. Finland/
25. France/
26. Polynesia/
27. Germany/
28. Gibraltar/
29. Greece/
30. Greenland/
31. Guam/
32. Hong Kong/
33. Hungary/
34. Iceland/
35. Ireland/
36. United Kingdom/
37. Israel/
38. Italy/

39. Japan/
40. "Republic of Korea"/
41. Kuwait/
42. Latvia/
43. Liechtenstein/
44. Lithuania/
45. Luxembourg/
46. Macau/
47. Malta/
48. Monaco/
49. Micronesia/
50. Netherlands/
51. New Caledonia/
52. New Zealand/
53. Micronesia/
54. Norway/
55. Oman/
56. Palau/
57. Poland/
58. Portugal/
59. Puerto Rico/
60. Qatar/
61. San Marino/
62. Saudi Arabia/
63. Seychelles/
64. Singapore/
65. Sint Maarten/
66. Slovakia/
67. Slovenia/
68. Spain/
69. "Saint Kitts and Nevis"/
70. st martin.mp.
71. Sweden/
72. Switzerland/
73. (trinidad and tobago).mp. [mp=title, abstract, original title, name of substance word, subject heading word, floating sub-heading word, keyword heading word, organism supplementary concept word, protocol supplementary concept word, rare disease supplementary concept word, unique identifier, synonyms]
74. (turks and caicos islands).mp. [mp=title, abstract, original title, name of substance word, subject heading word, floating sub-heading word, keyword heading word, organism supplementary concept word, protocol supplementary concept word, rare disease supplementary concept word, unique identifier, synonyms]
75. United Arab Emirates/
76. United States/
77. Uruguay/
78. United States Virgin Islands/
79. 1 or 2 or 3 or 4 or 5 or 6 or 7 or 8 or 9 or 10 or 11 or 12 or 13 or 14 or 15 or 16 or 17 or 18 or 19 or 20 or 21 or 22 or 23 or 24 or 25 or 26 or 27 or 28 or 29 or 30 or 31 or 32 or 33 or 34 or 35 or 36 or 37 or 38 or 39 or 40 or 41 or 42 or 43 or 44 or 45 or 46 or 47 or 48 or 49 or 50 or 51 or 52 or 53 or 54 or 55 or 56 or 57 or 58 or 59 or 60 or 61 or 62 or 63 or 64 or 65 or 66 or 67 or 68 or 69 or 70 or 71 or 72 or 73 or 74 or 75 or 76 or 77 or 78
80. Pregnancy/
81. Preconception Care/
82. periconceptional.mp.
83. pre pregnancy.mp.
84. Prenatal Care/
85. Perinatal Care/
86. Pregnant Women/
87. Folic Acid/
88. folvite.mp.
89. pteroglutamic acid.mp.
90. vitamin B9.mp.
91. vitamin M.mp.
92. unmetabolised folic acid.mp.
93. unmetabolized folic acid.mp.
94. folate.mp.
95. Food, Fortified/
96. food supplemented.mp.
97. Dietary Supplements/
98. Biofortification/
99. food supplements.mp.
100. Food Assistance/
101. Nutrition Surveys/

102. Health Surveys/
103. prenatal supplement.mp.
104. 80 or 81 or 82 or 83 or 84 or 85 or 86
105. 87 or 88 or 89 or 90 or 91 or 92 or 93 or 94
106. 95 or 96 or 97 or 98 or 99 or 100 or 101 or 102 or 103
107. 79 and 104 and 105 and 106
108. limit 107 to yr="1998 -Current"

☐ **Revised folate intake OR** Permanent

English Français Italiano Deutsch 日本語 繁體中文 Español 简体中文 한국어

About Us Contact Us Privacy Policy Terms of Use

© 2021 Ovid Technologies, Inc. All rights reserved. OvidUI_04.15.00.096, SourceID 206630d28626f3794d7f44308ce35cd7bc30c040
