# Supplementary material for: Women Taking a Folic Acid Supplement in Countries with Mandatory Food Fortification Programs May Be Exceeding the Upper Tolerable Limit of Folic Acid: A Systematic Review
Source: Nutrients. 2022 Jun 29;14(13):2715. doi: 10.3390/nu14132715 (PMC9268323; doi:10.3390/nu14132715)
Supplement: Supplementary file 1 [file nutrients-14-02715-s001.zip › Table S1 - Quality assessment of studies v1.pdf]

Table S1. Quality assessment of studies.

Study Name	1. Introduction - aims/objectives clear?	2. Methods - study design appropriate?	3. Sample size justified?	4. Target/reference pop. clearly defined?	5. Sample frame appropriate ?	6. Was the selection process appropriate?	7. Non responders categorised?	8. Risk factors/outcomes appropriate to the aims?	9. Risk factors measured correctly?	10. SI and/or precision estimates?	11. Methods described for repetition?	12. Basic data adequately described?	13. Response rate & non-response bias?	14. Non-responders described?	15. Were the results internally consistent?	16. Results presented for all the analyses?	17.Discussions conclusions justified by	18. Limitations ?	19. Funding sources/ conflicts?	20. Ethical approval/consent?
Yang 2007	Yes	Yes	Yes	Yes	Yes	Yes	Yes	Yes	Yes	Yes	Yes	Yes	No	UN	UN	Yes	Yes	Yes	No	Yes
Whitrow 2009	Yes	Yes	UN	Yes	Yes	Yes	Yes	Yes	Yes	Yes	Yes	Yes	No	Yes	Yes	Yes	Yes	Yes	UN	Yes
Trivedi 2018	Yes	Yes	UN	Yes	Yes	Yes	UN	Yes	Yes	Yes	Yes	Yes	No	UN	Yes	Yes	Yes	Yes	No	Yes
Tinker 2012 (obesity)	Yes	Yes	Yes	Yes	Yes	Yes	Yes	Yes	Yes	UN	Yes	Yes	No	UN	Yes	Yes	Yes	Yes	UN	Yes
Tinker 2012	Yes	Yes	Yes	Yes	Yes	Yes	UN	Yes	Yes	UN	Yes	No	No	UN	Yes	No	Yes	Yes	No	Yes
Sotres-Alvarez 2013	Yes	Yes	UN	Yes	Yes	Yes	UN	Yes	Yes	Yes	Yes	Yes	UN	UN	Yes	Yes	Yes	Yes	No	Yes
Shin 2016	Yes	Yes	Yes	Yes	Yes	Yes	Yes	Yes	Yes	Yes	Yes	Yes	No	Yes	Yes	Yes	Yes	Yes	No	Yes
Roy 2012	Yes	Yes	UN	Yes	Yes	Yes	Yes	Yes	Yes	Yes	Yes		No	UN	Yes	Yes	Yes	Yes	No	Yes
Rai 2015	Yes	Yes	Yes	Yes	Yes	Yes	Yes	Yes	Yes	Yes	Yes	No	No	UN	UN	Yes	Yes	Yes	No	Yes
Plumptre 2015	Yes	Yes	Yes	Yes	Yes	Yes	Yes	Yes	Yes		Yes	Yes	No	Yes	Yes	Yes	Yes	Yes	No	Yes
Pick 2005	Yes	Yes	UN	Yes	Yes	Yes	Yes	Yes	Yes	Yes	Yes	Yes	No	UN	Yes	Yes	Yes	Yes	No	Yes
Murphy 2021-Impact	Yes	Yes	UN	Yes	Yes	Yes	Yes	Yes	Yes	Yes	Yes	Yes	No	UN	Yes	Yes	Yes	Yes	No	Yes
Murphy 2021 - Gestational	Yes	Yes	UN	Yes	Yes	Yes	UN	Yes	Yes	UN	Yes	Yes	No	UN	Yes	Yes	Yes	Yes	No	Yes
Mojtabai 2004	Yes	Yes	Yes	Yes	Yes	Yes	Yes	Yes	Yes	Yes	Yes	Yes	UN	UN	Yes	Yes	Yes	Yes	UN	Yes
Masih 2015	Yes	Yes	Yes	Yes	Yes	Yes	Yes	Yes	Yes	Yes	Yes	Yes	No	Yes	Yes	Yes	Yes	Yes	No	Yes
Martinussen 2012	Yes	Yes	UN	Yes	Yes	Yes	Yes	Yes	Yes	Yes	Yes	Yes	No	UN	Yes	Yes	Yes	Yes	No	Yes
Marchetta 2016	Yes	Yes	Yes	Yes	Yes	Yes	Yes	Yes	Yes	Yes	Yes	Yes	No	UN	Yes	Yes	Yes	Yes	No	Yes
Livock 2017	Yes	Yes	Yes	Yes	Yes	Yes	Yes	Yes	Yes	Yes	Yes	UN	No	Yes	Yes	Yes	Yes	Yes	No	Yes
Jun-20	Yes	Yes	Yes	Yes	Yes	Yes	UN	Yes	Yes	Yes	Yes	Yes	UN	UN	Yes	Yes	Yes	Yes	No	Yes
Hure 2009	Yes	Yes	Yes	Yes	Yes	Yes	UN	Yes	Yes		Yes	Yes	UN	UN	Yes	Yes	Yes	Yes	No	Yes

Hromi-Fiedler 2012	Yes	Yes	UN	Yes	Yes	Yes	UN	Yes	Yes	Yes	Yes	Yes	UN	No	Yes	Yes	Yes	Yes	No	Yes
Hamner 2013	Yes	Yes	Yes	Yes	Yes	Yes	Yes	Yes	Yes	Yes	Yes	Yes	UN	Yes	Yes	Yes	Yes	Yes	No	Yes
Gomez 2015	Yes	Yes	Yes	Yes	Yes	Yes	Yes	Yes	Yes	Yes	Yes	Yes	UN	No	Yes	Yes	Yes	Yes	No	Yes
Gaskins 2012	Yes	Yes	UN	Yes	Yes	Yes	Yes	Yes	Yes	Yes	Yes	Yes	No	UN	UN	Yes	Yes	Yes	No	Yes
Gaskins 2019	Yes	Yes	UN	Yes	Yes	Yes	UN	Yes	Yes	Yes	Yes	Yes	UN	UN	Yes	Yes	Yes	Yes	No	Yes
Gaskins 2014	Yes	Yes	UN	Yes	Yes	Yes	UN	Yes	Yes	UN	No	Yes	UN	UN	Yes	Yes	Yes	Yes	No	Yes
Furness 2013	Yes	Yes	Yes	Yes	Yes	Yes	UN	Yes	Yes	Yes	Yes	Yes	No	UN	Yes	Yes	Yes	Yes	No	Yes
Dubois 2017	Yes	Yes	Yes	Yes	Yes	Yes	Yes	Yes	Yes	Yes	Yes	Yes	Yes	UN	Yes	Yes	Yes	Yes	No	Yes
Dorise 2020	Yes	Yes	Yes	Yes	Yes	Yes	UN	Yes	Yes	Yes	Yes	Yes	No	No	Yes	Yes	Yes	Yes	No	Yes
Dietrich 2005	Yes	Yes	Yes	Yes	Yes	Yes	Yes	Yes	Yes	Yes	No	Yes	No	Yes	Yes	Yes	Yes	Yes	UN	Yes
Crider 2018	Yes	Yes	Yes	Yes	Yes	Yes	Yes	Yes	Yes	Yes	Yes	Yes	No	Yes	Yes	Yes	Yes	Yes	No	Yes
Cena 2008	Yes	Yes	No	Yes	Yes	Yes	No	Yes	Yes	Yes	Yes	Yes	No	No	Yes	Yes	Yes	Yes	No	Yes
Boeke 2013	Yes	Yes	UN	Yes	Yes	Yes	No	Yes	Yes	Yes	Yes	Yes	No	Yes	Yes	Yes	Yes	Yes	UN	Yes
Beringer 2021	Yes	Yes	UN	Yes	Yes	Yes	UN	Yes	Yes	No	Yes	Yes	UN	UN	Yes	Yes	Yes	Yes	No	Yes
Bailey 2019	Yes	Yes	Yes	Yes	Yes	Yes	UN	Yes	Yes	Yes	Yes	Yes	UN	UN	Yes	Yes	Yes	Yes	Yes	Yes
French 2003	Yes	Yes	No	Yes	Yes	Yes	Yes	Yes	Yes	Yes	Yes	Yes	UN	No	Yes	Yes	Yes	UN	No	Yes

Legend: UN=unknown, SI= statistical significance
